# Costs of common perinatal mental health problems in South Africa

**DOI:** 10.1017/gmh.2022.48

**Published:** 2022-08-23

**Authors:** Annette Bauer, Emily Garman, Donela Besada, Sally Field, Martin Knapp, Simone Honikman

**Affiliations:** 1 London School of Economics and Political Science (LSE), Care Policy and Evaluation Centre, Houghton Street, London WC2A 2AE, UK; 2 Department of Psychiatry and Mental Health, Alan J Flisher Centre for Public Mental Health, University of Cape Town, Cape Town, South Africa; 3 Health Systems Unit, South African Medical Research Council, Cape Town, South Africa; 4 Department of Psychiatry and Mental Health, Perinatal Mental Health Project, Alan J Flisher Centre for Public Mental Health, University of Cape Town, Cape Town, South Africa

**Keywords:** Child impact, cost analysis, maternal mental health, perinatal mental health, South Africa

## Abstract

**Background:**

Perinatal mental health problems, defined as mental health problems occurring from the start of pregnancy to one year after birth, substantially affect women's and children's quality of life in low- and middle-income countries. In South Africa, despite high prevalence and documented negative impacts, most women do not receive any care.

**Methods:**

A modelling study examined the costs of perinatal mental health problems, namely depression and anxiety, for a hypothetical cohort of women and their children in South Africa over part of their life course (10 years for women, 40 years for children). In sensitivity analysis, additional impacts of post-traumatic stress disorder (PTSD) and completed suicide were included. Data sources were published findings from cohort studies, as well as epidemiological and economic data from South Africa. Data from international studies were considered where no data from South Africa were available.

**Results:**

Lifetime costs of perinatal depression and anxiety in South Africa amount to USD 2.8 billion per annual cohort of births. If the impacts of PTSD and suicide are included, costs increase to USD 2.9 billion. This includes costs linked to losses in quality of life (USD 1.8 billion), losses in income (USD 1.1 billion) and public sector costs (USD 3.5 million).

**Conclusions:**

Whilst important progress has been made in South Africa with regards to mental health policies and interventions that include assessment and management of perinatal mental health problems, substantial underinvestment prevents progress. Findings from this study strengthen the economic case for investing in perinatal mental health care.

## Background

Perinatal mental health problems, defined in this paper as maternal mental health problems during the period from the start of pregnancy to one year postpartum, are highly prevalent among women giving birth and contribute substantially to the global burden of disease (Fisher, 2011). In South Africa, estimates of the prevalence of perinatal depression, stress or anxiety range widely from 16% to 50% (Ramchandani *et al*., 2009; Rochat *et al*., 2011; Brittain *et al*., 2015; van Heyningen *et al*., 2016), reflecting measurement challenges and differences in population characteristics. Furthermore, 10% of women are at high risk of suicide during the perinatal period (Dewing *et al*., 2013; Rochat *et al*., 2013; van Heyningen *et al*., 2016; Garman *et al*., 2019a). This significant burden has been exacerbated by the coronavirus disease 2019 (COVID-19) pandemic (Abrahams *et al*., 2021).

In addition to causing substantial human suffering for women, there are well-established adverse impacts of untreated perinatal mental health problems on pregnancy outcomes, infant growth and development, and offspring educational attainments (Stewart, 2007; Stein *et al*., 2014; Glover *et al*., 2018). Evidence from South African studies shows negative impacts of postnatal depression or psychological distress on short- and long-term child outcomes, including respiratory tract infections, recurrent wheezing or asthma (MacGinty *et al*., 2018, 2019, 2020; Kariuki *et al*., 2020), stunting (Avan *et al*., 2010; Tomlinson *et al*., 2006), and mental health problems (Verkuijl *et al*., 2014). Risk factors include a complex combination of genetic, biological, social, psychological and environmental factors. For example, antenatal anxiety increases maternal cortisol concentrations, which have been associated with changes to foetal brain development (Stein *et al*., 2014; Glover *et al*., 2018). In the postnatal period, psychological factors may affect mothers' responsiveness to their infants and nurturing abilities (Stein *et al*., 2014). Poverty, domestic violence, addictions and lack of social support, especially from another caregiver or partner, worsen negative impacts on the child (Stein *et al*., 2014).

Globally and nationally, most perinatal mental health problems remain unidentified and untreated (Engle, 2009; English *et al*., 2017). While the urgent need to address perinatal depression as a key risk factor for child development has been highlighted by bodies such as the World Health Organization (WHO) (Fisher, 2011), this has not yet led to adequate policies or investments in most low- or middle-income countries. In South Africa, although important progress has been made with regards to mental health policies, such as the introduction of routine screening, and trialled pilots, including those that assess and manage common mental disorders during the perinatal period (Honikman *et al*., 2012; National Department of Health, 2012; Lund *et al*., 2014; Baron *et al*., 2016), underinvestment, under-resourcing and major implementation challenges have prevented progress and scaling-up (Docrat *et al*., 2019a; Honikman and Sigwebela, 2020).

Untreated perinatal mental health problems generate substantial human suffering and high costs (Bauer *et al*., 2016). In addition to quality-of-life losses, this includes women's reduced ability to pursue income-generating or other productive activities (Howard *et al*., 2014). Children's reduced quality of life and life-time earnings because of long-term health and development problems are likely to be substantial. There are also costs associated with increased hospital service use resulting from severe infant morbidities (Weobong *et al*., 2015; Jacques *et al*., 2019). For example, in the United Kingdom (UK), the costs of perinatal depression and anxiety were estimated to be £800 million (USD 1.1 billion) per 100 000 births (Bauer *et al*., 2016).

There have been very few economic studies of perinatal mental health problems, particularly in low- or middle-income contexts. The aim of this study is to address this gap in South Africa by examining the lifetime costs of perinatal mental health problems associated with the impacts on mothers and their children.

## Method

As done in an earlier UK study (Bauer *et al*., 2016), we applied decision-analytic modelling techniques (Briggs *et al*., 2006) to simulate the additional costs because of (untreated) perinatal mental health problems. A hypothetical cohort of women and their children were followed in annual cycles over time (10 years for women, 40 years for children) to cover the lifetime over which most impacts occur.

For the model parameters, we used: peer-reviewed publications of results from birth cohort studies such as the Drakenstein Child Health Study (Stein *et al*., 2015) and the Birth-to-Twenty (Bt20) cohort (Richter *et al*., 2004); as well as survey data on population, economic and health indicators published by national and international organisations (WHO, World Bank and South African government). Where we drew on international evidence or evidence not specific to the perinatal period, this is made explicit. All parameters used for the study, values, data sources, and details of how values were derived are presented in Table 1.

**Table 1.** Parameters, values, and data sources for the modelling

Parameter	Value	Data source and details
Model cohort (mothers)		
Number of live births (in 2017)	989 318	Stats South Africa 2017; http://www.statssa.gov.za
Probability of still birth	1.44%	Still birth rate of 14.4 per 1000 births (Lawn *et al*., 2016)
Number of women dying from maternal death (i.e. pregnancy related), antenatal period	350	Derived from number of maternal deaths in 2017 of 1400^1^ and prop. related to antenatal period of 25% (Kassebaum *et al*., 2014)
Number of women dying from maternal death (i.e. pregnancy related), postnatal period	1050	Derived from number of maternal deaths in 2017 of 1400^2^ and prop. related period over which maternal deaths are accounted i.e. 42 days after birth/ pregnancy termination (Kassebaum *et al*., 2014)
Annual probability of death (women aged 15 to 39)	0.42%	Global Burden of Disease Life Tables 2017^3^
Proportion of women with perinatal mental health problems		
Depressive symptoms meeting threshold of diagnosis, moderate + severe	20%	Estimated lower value from various studies
Depressive symptoms, severe v. moderate	8% v. 92%	Estimated from Global Burden of Disease study (Burstein *et al*., 2015)
Anxiety without depression	7.5%	South African study (Redinger *et al*., 2018)
Post-traumatic stress disorder (antenatal)	10%	South African studies (van Heyningen *et al*., 2017; Redinger *et al*., 2018; van Heyningen *et al*., 2018)
Post-traumatic stress disorder (postnatal)	5%	South African study (Redinger *et al*., 2018)
Proportional cause suicide in relation to all-cause mortality (perinatal period)	20%	International review (Lindahl *et al*., 2005)
Average duration of perinatal mental health problems (in yrs.)	0.25	Dutch study (Spijker *et al*., 2002); similar values suggested in South African study (Garman *et al*., 2019b)
Proportion of women with ongoing perinatal mental health problems (from 1 to 2nd year postpartum)		
Among those with depression meeting threshold for diagnosis or anxiety disorder	10%	Dutch study (Spijker *et al*., 2002); similar values suggested by South African study (Garman *et al*., 2019b); estimates refer to depression
Disability weights for maternal mental health problems		
Depressive symptoms, moderate	0.396	Global Burden of Disease study 2013^4^
Depressive symptoms, severe	0.658	Global Burden of Disease study 2013^5^
Value of DALY (GDP per capita, in 2017, USD)	12 703	World Bank, GDP data (including projections)^6^
Income decrement for women with severe depression or anxiety	67.5%	Derived from South African study (Lund *et al*., 2013)
Women's income, in 2017, USD	5519	Derived from World Bank, GDP data (including projections)^7^; 90% of average income to reflect gender pay gap e.g. South African study (Lund *et al*., 2013)
Model cohort (children)		
Number live births	989318	Stats South Africa 2017; http://www.statssa.gov.za
Annual probability of death	1st yr. 0.34%; 2nd yr. 0.17%; 3rd yr. 0.12%; 4th yr. 0.1%; 5th yr. 0.08%; 6–9th yrs. 0.07%; 10th to 14th yrs. 0.06%; 15th to 19th yrs. 0.1%; 20th to 24th yrs. 0.2%; 25th to 29th yrs. 0.5%; 30th to 34th yrs. 0.7%; 25th to 40th yrs. 0.8%	Global Burden of Disease Life Tables 2017^8^
Proportion children exposed to perinatal mental health problems		
Postnatal depression	20%	See above
Postnatal psychological distress	7%	(MacGinty *et al*., 2018, 2019, 2020)
Risk difference for child problems in excess due to exposure to diagnosable postnatal depression or long-term postnatal psychological distress		
Stunting (due to diagnosable postnatal depression)	15%	Derived from: (Tomlinson *et al*., 2006); exposed v. unexposed group 33% v. 18%
Externalising problems (due to diagnosable postnatal depression)	5%	Derived from: (Verkuijl *et al*., 2014); exposed v. unexposed group 14% v. 9%
Internalising problems (due to diagnosable postnatal depression)	4%	Derived from: (Verkuijl *et al*., 2014); exposed v. unexposed group 13% v. 9%
Severe lower tract respiratory infection requiring hospitalisation (due to long-term postnatal psychological distress)	14%	Derived from: (MacGinty *et al*., 2019); exposed v. unexposed group 32% v. 17%
Asthma (due to long-term postnatal psychological distress)	7%	Derived from: (MacGinty *et al*., 2018) exposed v. unexposed group 23% v. 16%
Disability weights for child problems		
Conduct disorder	0.241	Global Burden of Disease 2013^9^; conduct disorder
Emotional disorder	0.265	Global Burden of Disease 2013^10^; weighted average moderate depression and anxiety
Lower respiratory tract infection, severe	0.133	Global Burden of Disease 2013^11^; severe infection
Asthma	0.036	Global Burden of Disease 2013^12^; refers to partly controlled asthma
Unit costs		
Income decrement for the child with stunting per year	10%	Pooled estimate across five cohorts from five low- and middle-income countries including South Africa in study (Martorell *et al*., 2010)
Hospitalisation, in USD	910	South African study (Ramjee, 2013); refers to the cost per inpatient admission to the public hospital

1 https://data.unicef.org/topic/maternal-health/maternal-mortality/

2 Ibid

3 https://cloud.ihme.washington.edu/index.php/s/2JLHyPXCnZQyd9Q?path=%2FLife%20Tables

4 Ibid

5 Ibid

6 https://data.worldbank.org/country/south-africa

7 Ibid

8 https://cloud.ihme.washington.edu/index.php/s/2JLHyPXCnZQyd9Q?path=%2FLife%20Tables

9 Ibid

10 Ibid

11 Ibid

12 Ibid

First, we describe how we estimated the number of women who develop perinatal mental health problems and the children who develop problems resulting from their exposure.

### Focus on anxiety and depression

We focused on common perinatal mental health problems, namely depression and anxiety, for which there was sufficiently robust prevalence data, based on diagnostic measures, differentiated into ante- and postnatal periods. Because data on prevalence vary widely between studies in different settings, conservative estimates were chosen. Since prevalence data were chosen based on diagnostic measures, mild conditions that do not meet the disease threshold were excluded. For both ante- and postnatal depression, a rate of 20% was chosen for the analysis. From international data, we applied proportions of 92% and 8% for moderate and severe depression, respectively (Burstein *et al*., 2015). For anxiety, an adjusted prevalence was estimated to reflect women without co-existing depression (and, for simplicity, no further distinction is made regarding severities). Since a study in Johannesburg found that, amongst 15% of women with probable anxiety disorder, half have co-existing probable depression (Redinger *et al*., 2018), a prevalence of 7.5% of anxiety was taken. Estimates align with a study from Cape Town that employed a diagnostic measure for anxiety disorder and excluded moderate depression or post-traumatic stress disorder (PTSD), reporting the prevalence of 8% (van Heyningen *et al*., 2017). In the absence of data for anxiety for the postnatal period, we assumed an equal prevalence, as indicated in other studies (Dennis *et al*., 2017).

### Estimating a hypothetical cohort of affected women and children

Numbers of mothers in the cohort were estimated based on number of live births reported in national statistics and prevalence data for perinatal depression and anxiety (noted above). We included all women aged 15 to 39 years who gave birth in 2017. This is the age range when most women or girls give birth. To estimate the economic impact of perinatal mental health problems during the antenatal period, adjustments were required to include women with still-births. For simplicity, no adjustments were made to reflect multiple births (e.g. twins) since this figure is very small. In line with the UK study (Bauer *et al*., 2016) the chronic nature of the problems for some women was captured by modelling for a proportion of these women who have persistent perinatal mental health problems for a 10-year period post-birth. Data were taken from a longitudinal study in a township near Cape Town (Garman *et al*., 2019b) which showed that, for about 10% of women with postnatal depression, the condition is chronic. We estimated that the condition lasts for up to 10 years. Starting from the first year after birth, we modelled a yearly, linear reduction of women with chronic perinatal depression or anxiety to reflect natural remission (Bauer *et al*., 2016). The same proportion was assumed and calculations applied for postnatal anxiety (Bauer *et al*., 2016).

The cohort number was reduced each year by the proportion of women who die over this period, based on age- and gender-specific mortality data from national statistics. For the probability of death during the perinatal period, we applied an additional mortality risk attributed to pregnancy- and maternity-related causes (see Table 1).

To estimate the additional number of children who develop adverse outcomes linked to their exposure, the number of live births was multiplied by the prevalence of perinatal mental health problems and the additional risk that children develop problems as a result. The latter is derived from birth cohort studies in South Africa that identify significant effects of perinatal mental health problems on: severe lower respiratory tract infections in the first year after birth (MacGinty *et al*., 2019), asthma (MacGinty *et al*., 2018), internalising problems (defined as being anxious or depressed), externalising problems (defined as showing aggressive or rule-breaking behaviour) (Verkuijl *et al*., 2014), and stunting (Tomlinson *et al*., 2006, 2018).

Specifically, numbers of children with internalising or externalising problems or stunting linked to perinatal mental health problems were estimated from the number of women with perinatal depression. Numbers of children with lower respiratory tract infection in the first year or ongoing asthma (which is linked to perinatal psychological distress) were estimated from the number of children exposed to perinatal psychological distress (which overlaps with depression and anxiety) based on work by MacGinty and colleagues (MacGinty *et al*., 2018, 2019). Evidence from that study indicated that lower respiratory infections occur only in the first year, whilst asthma develops as a long-term condition and persists over the lifetime (i.e. 40 years in this model).

As done for mothers, we modelled children in the cohort who are alive each year by applying mortality probabilities derived from national statistics.

### Estimating economic consequences

Disability weights and unit costs (multiplied by assumed disease durations) were assigned to adverse impacts for mothers and children. Our approach follows recommendations by the WHO to include costs borne by both government and wider society (WHO, 2003). Here, government costs refer to the use of publicly-funded healthcare care for children admitted to hospital because of severe lower respiratory tract infections in the first year after birth. We were unable to identify any other government costs directly applicable to the population. Societal costs included productivity losses and losses in quantity and quality of life, measured in the form of disability-adjusted life years (DALYs). Valuing both productivity and DALYs separately followed an approach in which (mental) health is valued distinctively for its contribution to labour force participation and its contribution to other intrinsic benefits (e.g. ability to enjoy life or to contribute to society such as performing parenting or caring tasks). We followed the approach proposed by the World Bank and WHO (Chisholm *et al*., 2016; Stenberg *et al*., 2016), and applied values equal to per capita gross domestic product (GDP) to income gains or losses and 0.5 per capita GDP to a DALY. Per capita GDP data were taken from World Bank estimates (https://data.worldbank.org), which include the impact of COVID-19 on GDP for 2020. For simplicity, and to reflect an uncertain outlook for economic recovery in South Africa, we applied 2020 GDP values for subsequent years.

Table 2 summarises the economic consequences included in the modelling and sources of cost data.

**Table 2.** Economic consequences included in the analysis

Outcome	Economic consequences	Data
Maternal mental illness during perinatal period (antenatal + postnatal depression/anxiety/post-traumatic stress disorder/ suicide)	Health impact (Disability-adjusted life years, DALYs)	Global Burden of Disease study; disability weights
	Income	Data on link between mental illness and income, adjusted for women
Chronic depression and anxiety linked to episode during the perinatal period (up to 10 years)	Health impact (Disability-adjusted life years, DALYs)	Global Burden of Disease study; disability weights
	Income	Data on the link between mental illness and income, adjusted for women
Child stunting	Income	Economic returns to education data
Child mental health problems	Quality of life (Disability-adjusted life years, DALYs)	Global Burden of Disease study; disability weights
Child lower respiratory tract infection, first year after birth	Quality of life (Disability-adjusted life years, DALYs)	Global Burden of Disease study; disability weights
	Public sector cost	Unit cost for hospitalisation
Child asthma	Quality of life (Disability-adjusted life years, DALYs)	Global Burden of Disease study; disability weights

#### Economic consequences: mothers

Economic consequences linked directly to mothers are losses in quantity and quality of life (measured by DALYs) and in productivity.

DALYs were calculated based on the number of women with depression or anxiety, disability weights and a duration of 3 months over which the problems last (Garman *et al*., 2019b). Disability weights for moderate and severe depression were applied to the respective numbers of mothers with those symptom severities, while for anxiety disorders, a disability weight for moderate anxiety was applied for all women with anxiety (since the model did not make further distinctions for severity levels).

Women's productivity losses were calculated based on the estimated annual income decrement for women with severe depression (Lund *et al*., 2013) applied to the estimated number of women with severe depression during and after the perinatal period. Findings from that study suggest that the income of women with severe depression is a third of the income that women *without* severe depression can expect to earn in South Africa. This value was applied using GDP per capita data, adjusting for a lower value for women (Lund *et al*., 2013).

#### Economic consequences: children

Economic consequences linked to children are losses in quantity and quality of life (measured by DALYs), productivity losses (linked to stunting) and hospital care (linked to severe lower respiratory tract infections in the first year after birth). Effects on DALYs were calculated for the additional number of children who experience severe lower respiratory tract infections in the first year after birth, asthma, or internalising or externalising mental health problems. Disability weights were for: severe infections to reflect severe lower respiratory tract infections; partly controlled asthma to reflect asthma; a weighted average for moderate depression or anxiety to reflect internalising problems (there is no disability weight for internalising problems as such); moderate conduct disorder to reflect child externalising problems. There is no disability weight for stunting and so we did not include this in the calculation of quantity or quality of life losses.

Depending on the data, different assumptions were made about disease durations or persistence of health impacts. The impact of children's mental health problems was assumed to persist for 40 years, a figure supported by national longitudinal study data up to 10 years later (Verkuijl *et al*., 2014) and several larger cohort studies from high-income settings which follow children until they are young adults (Pearson *et al*., 2013; O'Donnell *et al*., 2014; Rajyaguru *et al*., 2021). As described above, for asthma, we also assumed long-term persistence (i.e. 40 years) due to the chronic nature of the condition, whilst for severe lower respiratory tract infections, only the initial episode during the postpartum year was considered.

Productivity losses linked to one child outcome – stunting – were calculated for the period starting from when children reach age 16 years, which is the minimum age for admission to employment in South Africa (https://www.labourguide.co.za). The strong negative links between stunting, lost school years and earnings – and the impact of perinatal depression on these outcomes – are well established from previous studies (Martorell *et al*., 2010; Smith Fawzi *et al*., 2019). An annual income decrement (Martorell *et al*., 2010) was applied to the additional number of children who are stunted linked to postnatal depression, taken from pooled data across five cohorts in low- and middle-income countries, including South Africa (Martorell *et al*., 2010).

Costs to the public sector refer to costs of hospitalisations of infants with episodes of severe lower respiratory tract infections linked to persistent psychological distress during the first year postpartum, calculated from South African evidence on hospitalisation risk (MacGinty *et al*., 2018, 2019) and data on the average cost of a hospital stay (Ramjee, 2013).

### Discounting

Costs incurred in the future are valued less than the same costs incurred today, which is why economic studies apply a discount rate to future costs (and benefits). In keeping with global methods (Chisholm *et al*., 2016), we determined the present value of costs (in 2017) using a discount rate of 3% for DALYs, income losses and healthcare costs. Estimates therefore reflected the present and future costs as they occur for a hypothetical cohort of women giving birth in the present year (here: 2017) and the children they give birth to. The year 2017 was chosen as the ‘present year’ as this is the latest year for which most national statistical data were available.

### Sensitivity analysis

Sensitivity analysis was used to explore the uncertainty of values by analysing the impact of changing parameters in a model on the final results. For this sensitivity analysis, we included quality and quantity of life impacts linked to one additional perinatal mental health problem, PTSD, excluded from the main analysis because some parts of the costs were likely to be included as part of depression or anxiety. We also included quality and quantity of life impacts linked to (completed) suicide during the perinatal period only in sensitivity analysis, as reliable data on suicide are particularly difficult to obtain in low- and middle-income countries (Fuhr *et al*., 2014) and suicide is not invariably related to mental health problems (Onah *et al*., 2017).

Proportions of women with PTSD were estimated at 10% for the antenatal period (Koen *et al*., 2017; van Heyningen *et al*., 2017; MacGinty *et al*., 2020) and 5% for the postnatal period (MacGinty *et al*., 2018). DALYs were calculated by applying a disability weight for severe anxiety and a duration of 3 months. For women completing suicide during the perinatal period, we estimated DALYs based on a suicide rate among women of 4.5 per 100 000 (Matzopoulos *et al*., 2015) and a weight for years of life lost over the lifespan for someone dying during their mid-life taken from the Global Burden of Disease study.

## Results

The main results of the analysis are presented in Table 3. The costs of perinatal mental health problems are shown in the form of the average cost per woman giving birth, as well as across the estimated 1 000 053 pregnant women in 2017. Reported values reflect present (i.e. discounted) values of the costs as they occur over the lifespan for the hypothetical 2017 cohort of women, and the children they gave birth to, in that year. As described above, the ‘lifespan’ refers to a time horizon of ten years for the women's cohort and of 40 years for the children's cohort. Findings from the main analysis reflect the costs of perinatal anxiety and depression. Findings from sensitivity analysis reflect the combined costs of PTSD or completed suicide during the perinatal period in addition to the costs of perinatal anxiety and depression. The additional costs only include those that relate to mothers' quality of life since we were not able to find published studies that measured the impact of PTSD or completed suicide on children.

**Table 3.** Costs of perinatal mental health problems per woman giving birth and per cohort of women giving birth (based on 2017 data, in USD), findings from base case and sensitivity analysis

	Findings from main analysis: costs of perinatal depression and anxiety		Findings from sensitivity analysis: costs of perinatal depression, anxiety, post-traumatic stress disorder & suicide	
	Per woman giving birth	Per cohort in 000's	Per woman giving birth	Per cohort in 000's
Mother				
DALY loss	404	403 601	553	550 686
Income loss	873	864 076	873	864 076
Total cost mother	**1277**	**1 267 677**	**1426**	**1 414 762**
Child				
DALY loss	1372	1 356 990	1372	1 356 990
Income loss	157	155 732	157	155 732
Hospital costs	1.2	1221	1.2	1221
Total cost child	**1530**	**1 513 942**	**1530**	**1 523 847**
Total cost (mother + child)	**2808**	**2 791 524**	**2808**	**2 938 609**

Total societal costs of perinatal depression and anxiety in South Africa are USD 2.8 billion if the impacts of PTSD and completed suicide are excluded, and USD 2.9 billion if they are included. This includes costs linked to losses in quality of life of USD 1.8 billion (USD 0.4 billion for mothers and USD 1.4 billion for children) and to losses in income of USD 1.1 billion (USD 0.9 billion for mothers and 0.2 billion for children). Total societal costs are USD 2818 per woman giving birth if the impacts of PTSD and completed suicide are excluded and USD 2966 if they are included. The share of total costs falling on the public (health) sector is comparatively small: USD 3.5 million for the cohort, linked entirely to short-term costs for hospitalisations of the infant. Over 77% of the costs of reduced quality of life are linked to the child, whilst 85% of the costs of lost income are linked to the mother. If it is assumed that costs would occur at the same rate over a 40-year period, then total costs calculated here would be equivalent to a yearly cost of USD 0.12 billion for the total population in South Africa and USD 122 per child born per cohort of women and children.

## Discussion

Lifetime costs linked to perinatal mental health problems in South Africa are very high. If one considers the impacts of both perinatal depression and anxiety on women (for 10 years) together with the impacts on their children (for 40 years), these costs amount to USD 2.8 billion per cohort of women giving birth *each year*. Costs increase further when PTSD and completed suicide impacts are considered. To our knowledge, this is the first study to assess the long-term and intergenerational costs of perinatal mental health problems in South Africa. The study established the costs in one African country, which was feasible because relevant data sources were available, including data from large, well-conducted birth cohort studies. Whilst similar prevalence and impacts are likely to occur in other African countries, a lack of data currently prevents estimating those. The methodology employed here would be applicable to other countries where the necessary data are available and would thereby help to make the case for investment.

Our study has several strengths. By taking a long-term and intergenerational perspective on costs, it addresses a gap that commonly occurs because studies only consider the short-term costs to individuals with the condition, a limitation that has been highlighted by the WHO (Chisholm *et al*., 2016). They therefore underestimate the implementation challenges of moving from evidence to better policy and practice (Knapp and Wong, 2020). Our study is largely based on country-specific data, thus seeking to be relevant to decision-makers nationally. For example, whilst an independent impact of antenatal depression on pregnancy outcomes, such as low birth-weight and preterm birth, has emerged consistently from studies and meta-analyses globally (Dadi *et al*., 2020), this link could not be confirmed in South Africa (Sania *et al*., 2017; Christodoulou *et al*., 2019) and is therefore not included in the analysis. This country-specific approach is only possible because of the several longitudinal studies that have been conducted in South Africa, following mothers and their children from pregnancy or childbirth over time.

Our study also has several limitations, many of which relate to gaps in the evidence typical for modelling studies of this kind (Chisholm *et al*., 2016). It is difficult to establish accurate prevalence data for perinatal mental health problems, which vary substantially between regions and communities. Our conservative approach to prevalence estimates may have mitigated against these problems but may also have led to the underestimation of impacts. For example, the study underestimated the impacts of psychological distress that are below thresholds for diagnostic categorisation. There are other reasons why our estimates are likely to be below actual costs. For example, to avoid double-counting, we probably underestimated lost productivity linked to children's problems since we only valued productivity losses linked to an increased probability of stunting and not those linked to mental health problems.

Several economic impacts could not be included due to a lack of data, including impacts on fathers, families and communities, as well as impacts of more severe conditions like psychosis. The latter, whilst rare, are known to have substantial impacts on individuals, families and communities (Farooq, 2012). In addition, no data were available that would have allowed us to quantify the costs of using health services for mothers and children beyond first year postpartum. Additionally, we were also unable to include costs of treating injuries or physical health conditions associated with perinatal mental health problems (Johannsen *et al*., 2020), or the cost of attempted suicide. For example, even though evidence suggests that maternal depression during the perinatal period is linked to missed health visits, reduced adherence to immunisation schedules and reduced adherence to HIV treatment (Cook *et al*., 2018), we were unable to include these impacts. Similarly, we were unable to include potentially substantial hospital costs linked to attempted suicide (Benedict *et al*., 2020), for example when women access trauma units. At the same time, the low public sector costs we found in this study reflect the substantial underinvestment in publicly-funded maternal and child mental health services, as well as in publicly funded services more generally, including those required to support children with stunting and mental health problems in order to prevent long-term disability and income losses.

We used data from studies that are unable to determine causality between perinatal mental health problems and negative child impacts. Even though studies sought to eliminate the influence of factors such as socio-economic status, HIV, co-existing physical health conditions, substance misuse or intimate partner violence, it cannot be fully ruled out that costs in our study related to the impacts of co-occurring factors. The importance of addressing social determinants of mental health problems, to prevent or reduce the long-term impact of mental health problems, is widely recognised (Lund *et al*., 2018).

Notwithstanding the limitations of modelling studies like this, findings from our study are an important part of the economic case for investment in this area and can be used to inform national policies and public financing decisions. Supporting the credibility of the results, assumptions were made following a conservative approach throughout, thus underestimating, rather than overestimating results.

Results from this study are consistent with previous findings. It has been estimated that the yearly costs of depression and anxiety among the general adult population globally amount to USD 1.15 trillion (Chisholm *et al*., 2016), and that the costs of perinatal depression linked to just a single economic impact, child stunting, for 137 low- and middle-income countries, are USD 14.5 billion (Smith Fawzi *et al*., 2019). When compared with findings from our cost study conducted in the UK (Bauer *et al*., 2016), which calculated costs of USD 7.8 billion for perinatal depression and anxiety, which is an equivalent to about USD 1.2 billion per 100 000 births, costs in South Africa are about a quarter of these costs. However, given that GDP per capita in South Africa is about a sixth of that in the UK, relative costs of perinatal mental health problems as a proportion of the GDP per capita are relatively much higher in South Africa (about 43% *v.* 28% in the UK in 2019) suggesting a relatively higher burden of perinatal mental problems. This is supported by evidence of the higher prevalence and more substantial health and economic impacts of mental health problems in low-and middle-income countries compared with high-income countries (Howard *et al*., 2014; Chisholm *et al*., 2016; Dadi *et al*., 2020).

Action is needed now to reduce the large human and economic costs of mental health problems to the population, which have been increasing as a result of the COVID-19 pandemic. This action should draw on local evidence for sustainable mental health financing strategies (Docrat *et al*., 2019b), national and international policies, such as the National Mental Health Policy Framework and Strategic Plan (Department of Health, 2013), South African Maternal, Perinatal and Neonatal Health Policy (Department of Health, 2021), WHO guidance on maternal mental health and early child development (WHO, 2008a, 2014, 2020) and by reference to the UN Sustainable Development Goals (WHO, 2008b). Intersectoral, collaborative and community-based strategies are needed to promote perinatal mental health by addressing social determinants of mental illness (such as gender-based violence, poverty, social isolation and obstetric violence), reducing mental health stigma, increasing demand for and promoting the uptake of care (Rahman *et al*., 2013, Atif *et al*., 2015, Lund *et al*., 2018, Macnab *et al*., 2022). Implementation strategies should include competency-based training, supervision and support for maternity staff and other frontline providers to provide primary-level mental healthcare (Honikman *et al*., 2012, Lund *et al*., 2014, Howard *et al*., 2014, WHO, PEPFAR & UNAIDS, 2007). Linked to this, investments should be made to create remunerated staff positions for non-specialist mental health providers in maternal and child service and community-based settings (Jacobs *et al*., 2021; Karyotaki *et al*., 2022; Patel, 2022).

## Conclusion

This study demonstrates the substantial costs to women, their children and to society of unaddressed perinatal mental health problems in South Africa. To avert ongoing costs, sustained investment is required to address perinatal mental health problems. Future research on the return on investment of scaling evidence-based interventions would yield further valuable data for government decision making.
